# Soybean Replacement by Alternative Protein Sources in Pig Nutrition and Its Effect on Meat Quality

**DOI:** 10.3390/ani13030494

**Published:** 2023-01-31

**Authors:** Silvia Parrini, Chiara Aquilani, Carolina Pugliese, Riccardo Bozzi, Francesco Sirtori

**Affiliations:** Department of Agriculture, Food, Environment and Forestry—DAGRI, University of Florence, 50144 Florence, Italy

**Keywords:** fava bean, pea, algae, insect meal, fatty acids, pork, swine, performance, feed

## Abstract

**Simple Summary:**

Soybean is the most common feed ingredient to supply protein to pigs. However, its production is linked to several issues related to environment (e.g., deforestation, water waste), production and transportation costs, as well as to ethical aspects including feed/food competition for land. In recent decades, research has endeavored to find alternatives to soybean in livestock feeds, especially for pigs. However, replacing this ingredient partially or totally may have consequences for animal growth rates, carcass yield and meat quality. The aim of this work was to review the research carried out in the last 10 years on this topic, identifying the alternative protein sources and their effects on pigs’ growth, carcass yield, and meat quality. Most of the ingredients recently studied are vegetable resources, especially local species of legumes well-adapted to specific climatic conditions. Moreover, some studies evaluated, as protein sources, oilseed by- and co-products, distillers’ dried grain with solubles, fish and animal proteins, and other co-products derived from other supply chains. Among innovative protein sources, microalgae and insects are gaining attention.

**Abstract:**

Soybean is one of the most expensive and limiting feed ingredients in diet formulations; however, in pig farming, it represents the main source of protein. The production and supply of soybean are critical steps due to their environmental impact and feed/food competition for land use. Therefore, research is focusing on finding alternatives to replace soybean partially or totally. However, alternative ingredients should ensure similar growth performance, carcass traits, and meat quality characteristics compared to conventional soybean-based diets. The objective of this review was to evaluate the impact of different alternative protein sources to soybean in pig nutrition and their effects on growth performance, carcass, and meat quality traits. The review process was performed on Scopus^®^, and it considered research findings published from 2012 to the present on the *Sus scrofa* species. Articles without a control group fed with soybean were discarded. The main alternative protein sources identified were other legumes and distillers’ dried grain with solubles (fish and animal proteins, oilseed by- and co-products). Interesting innovative protein sources included by-products from other industries (residues), microalgae and insects. Nevertheless, in dietary formulations, close attention must be paid to address the nutritional requirements, balance the supply of amino acids, avoid anti-nutritional or toxic compounds occasionally present in alternative protein sources, as well as determine the availability of protein feed in specific geographical areas.

## 1. Introduction

In livestock systems, proteins feeds are one of the most expensive and limiting feed ingredients in diet formulations [1]. The production and supply of feeds are critical steps due to the environmental impact they are connected to, such as land use change [2], land occupation [3], and energy and water use [4].

By 2050, the world population will reach more than 9 billion people, and consequently, agricultural production will need to increase by 50% to meet food demand [5,6], whereas the arable land per person is expected to decrease [7]. Simultaneously, the improvement of living standards in developing countries will determine an increase in global demand for sustainable animal protein [8,9].

At the European level, the environmental implications are related to the “protein gap”, which in turn has led to the importation of large amounts of raw protein materials for feeds and resulted in higher feed shipping costs [7]. In 2019, the industrial component of feed production was estimated at 1126 million tons, constant with respect to 2018, while an increase of 3% was recorded compared to 2017 [8]. The main imported protein feed is soybean (SB) due to the limited cultivated area available in Europe. The growth in SB demand is causing land use change worldwide, and it was directly linked to deforestation in South America, which increased feed’s carbon footprint [8]. Despite the impact of SB cultivation on tropical forest ecosystems [9] and the increasing awareness of the unsustainability of feeding livestock with diets based mostly on imported feed proteins [10], SB is still the first choice among protein feeds due to both its quality and its accessibility. Especially for monogastric (pigs and poultry) animals, post-extraction soybean meal (SBM) represents the main source of protein [11,12]. Eventually, feeding livestock with proteins also used by humans may worsen feed/food competition and impact food security [13]. Poultry and swine require ~42% of the total in feed production and supply 20% of animal proteins, while ruminants use about 19% of feed to produce 20% of animal protein [13]. Considering the organic pig sector, the difficulty in meeting demand for protein appears even more evident [14]. Further, some Protected Designation of Origin (PDO) products of relevant interest for European consumers impose specific rules for animals diets, including limitations on the supply of protein resources [15,16].

This causes a dependency on imports of non-genetically modified raw feeds that are more expensive than other resources [17,18]. Therefore, research on the possibilities of replacing SB in the nutrition of monogastric animals, particularly pigs, poultry and waterfowl, has been performed [19,20,21,22], also with attention to local or available feed resources for food production [23].

High protein sources can derive from conventional feed, including cereals, distillers’ dried grain with solubles (DDGS), legumes, fish and animal subproducts, oilseed (rapeseed meal; RM) and their respective co-products, biofuel coproducts, and amino acid formulations. Alternative feed protein sources also include by-products from other industries (residues), microalgae, insects, and single-cell organisms [24]. Nevertheless, numerous factors should be taken into consideration for interfering with or limiting the use of plant-based protein supplements, above all in young pigs [25]. Possible limitations for the use of plant proteins are their secondary plant constituents, some of which show anti-nutritional properties. However, these substances can be reduced thanks to successful innovations in feed technology: fermentation and enzymatic treatment, as well as the integration of exogenous enzymes [26].

Different diet formulations and protein sources during fattening can affect growth performance as well as carcass and meat quality traits. The aim of the present review was to examine the most recent research on alternative protein feeds to totally or partially replace SB in pig production and to identify the effects of these new dietary formulations on growth performance and carcass and meat quality traits.

## 2. Materials and Methods

An extensive literature screening was performed to evaluate the effects of replacing SB with alternative protein sources on slaughtering and meat quality traits of pork. The review process considered research findings published from 2012 to the present and was limited to the *Sus scrofa* species. Any geographical limitation was set. The literature search was performed on Scopus^®^ database on previously selected search strings, which were the following: “insect meal AND pig”, “seaweed AND pig”, “macroalgae AND pig”, “microalgae AND pig”; “soybean AND replacement AND pig”, “animal by-products meal AND pig”, “legumes AND pig”, “alternative protein AND feed AND pig”, “agricultural by-products AND protein AND pig”. A further search was performed by replacing “pig” with “pork” in the same strings. A total of 723 articles were found according to the keywords used. A first selection process was based on title and abstract examination to reduce the number of articles to be downloaded. Secondly, the selected articles were evaluated according to their adherence to the aim of the present review, e.g., discarding articles without a control group fed with SB, or whose aim was not to evaluate the effects of replacing SB on growth performance, carcass and/or meat quality traits. At the end of the screening, the articles were used to discuss the topic of interest.

## 3. Characteristics of Alternative Feed Resources Used in Pig Rearing 

The protein sources for feeding piglets and growing pigs can be classified by their origin, local availability, sustainability, environmental impact, and level of innovation (i.e., conventional or studied for decades or innovative). When evaluating the potential replacement of SB, it is necessary to consider the physical and chemical characteristics of alternative resources, the level of essential amino acids, and the presence of anti-nutritional factors, as well as how they are converted by animals’ physiology into final products. In this review, alternative protein resources were divided into two broad categories: plants and other (no plants). 

In many cases, different protein sources were used in combination, and in some cases also, amino acid addition was applied. The use of diets containing combinations of multiple protein sources to overcome their singular limits has been reported by different authors [24,25].

### 3.1. Plant Protein Resources

#### 3.1.1. Legume Alternatives to Soybean

Legumes, and in particular, dry seeds of species belonging to the Fabaceae family, have been used in feed formulations for pigs to complement cereals thanks to their chemical and physical characteristics. However, integration of essential amino acids is usually necessary to achieve balanced diets. Legumes show high protein content and low fat [27], but their nutritional composition is very different [28] among species and dependent on variety, location, growing conditions and management where they are cultivated [29]. Nevertheless, legume use in animal feeding is also limited by the presence of antinutritional components that may decrease growth rates, feed intake and feed utilization. Antinutritional compounds encompass alkaloids, flavonoids, glycosides, isoflavones, phenols, phytosterols, phytic acid, protease inhibitors, saponins, and tannins [28]. Non-processed and/or processed legume seeds have the highest potential to replace SB, avoiding the presence of GMO in feed mixtures for monogastric animals [12]. They are also a cheap energy and amino acid source in cereal-based diets. Despite the potential use of legumes in animal nutrition, in Europe (2016) the percentage of arable land dedicated to grain legumes was only 1.5% compared with 14.5% globally [30], but during the last 15 years, the areas cultivated in some legumes, such as *Pisum sativum* and *Vicia faba*, have been increasing [29]. In the swine sector, most of the studies in recent years considered a singular source [29,31,32] or in association with other raw materials (legumes and other vegetable resources) [18,33,34] to replace SBM completely or partially. The main legume resources used as alternatives to SB in pigs’ diets formulation are:

- Pea (*Pisum sativum* L.) (P), has high amino acid availability, in particular lysin and arginine, and low levels of anti-nutritional factors such as tannins and trypsin inhibitors, but it contains poorly digestible fiber for monogastrics [35] and low levels of methionine and other sulfur amino acids. In terms of environmental impact, the cultivation of peas in rotation systems requires a reduced use of inputs of nitrogenous fertilizer and, therefore, is associated with lower production costs [36].
- Fava bean (*Vicia faba* L.), (FB), also known as broad bean or field bean, is rich in protein (28.2% DM), carbohydrates (45.7 to 70.1% DM) and minerals (potassium, phosphorus, iron, and zinc) compared to general grain sources [37]. FB contains about 1.7% DM of fats, which are rich in major unsaturated fatty acids (i.e., oleic, palmitoleic and linoleic acids), while among its saturated fatty acids, palmitic and stearic acids represent the majority [38]. Regarding amino acid availability, FB seeds contain mainly arginine and leucine and high levels of lysine, and consequently could be mixed with cereals to achieve a balanced amino acid profile in monogastrics [39,40]. Low levels of tryptophan and methionine have been reported [41]. FB use in pig nutrition is limited due to the presence of antinutritional factors such as total phenolics, tannins, lectins, and protease inhibitor and trypsin inhibitor activity [25,38]. Dehulling, germination, soaking and thermal treatments have been applied to FB in order to reduce antinutritional factors or improve the intake and digestibility of nutrients [20,42]. Moreover, in the last decades, low-tannin varieties of FB have been introduced in the market [31]. Concerning the environmental impact, FB is a versatile legume produced in different climatic zones and diverse soils with a high capability for nitrogen fixation and consequent reduced need for nitrogen fertilizer [43].
- Lupin seed (*Lupinus* L.) is scarcely used in pig diets due both to its low palatability and to the presence of antinutritional factors such as alkaloids and non-starch polysaccharides and oligosaccharides that affect the nutritional characteristics and digestibility as well as the physiological mechanisms of the intestinal tract [1,28,29]. Nevertheless, lupin is high in protein content, ranging from 32 to 52%, depending on the variety [35]. Lupin is characterized by its favorable lysine and arginine content, but low content of methionine, tryptophan and threonine [29]. The hull of lupin represents about 15–30% of the seed weight. It contains cellulose that represents a limitation for monogastrics; consequently, dehulling can improve the utilization of kernels, while hulls could be used in ruminant species nutrition [1]. Different treatments such as germination, grinding, extrusion and thermal processing for micronization have been applied to lupin whole seeds to improve the nutritional value [28,44]. Nevertheless, the literature on the use of lupins is dated, even if lupin cultivars with low-alkaloid content have been recently reported for animal feed [29]. Yellow lupin (*Lupinus luteus*) seems to be preferred by pigs over narrow-leafed or albus lupins [35].
- Other legume species such as vetch (*Vicia sativa and Vicia narbonensis*), red peas (*Lathyrus cicera* and *L. sativus*), and ervil (*Vicia ervilia*) are less used in pig diets due both to their poor palatability and their antinutritional factors. Common vetch’s crude protein content averages 28.6% on a DM basis, although the range of variation between different varieties is large [45]; it is rich in minerals, has high energy content, and can potentially be used as a substitute for SB [45]. Nevertheless, the reduced use of common vetch seeds is due to the presence of several substances, such as c-glutamyl-b-cyano-alanine and b-cyano-L-alanine, that are toxic to monogastrics and also of antinutritional factors such as vicine, phenolics, trypsin inhibitor, tannins and convicin, which affect the bioavailability and absorption of nutrients [46]. For these reasons, the studies on the use of vetch in pig diets are very limited, although vetch has good sustainability in cultivation and seems to be less costly than alternatives [45]. At the global level, there are also some examples of using tropical forage legumes for pigs. Indeed, they represent a resource for specific territories, especially in smallholder production with limited access to other feeds.
- Tropical forage legumes such as psophocarpus (*Psophocarpus scandens*), stylosanthes (*Stylosanthes guianensis*) and vigna (*Vigna unguiculata*), thanks to their high protein value and energy digestibility, have been positively included in pigs’ diet formulations, as reported by Kambashi et al. [47]. In addition, leaf meals such as stylo (*Stylosanthes guianensis*) associated with porcupine joint vetch (*Aeschynomene histrix*) have been used to replace SB in the diets of Moo Lath Lao pigs. Stylo (*Stylosanthes guianensis* Aubl. Sw. var. *guianensis*), native to South America, is the legume most known among alternative protein sources available in certain geographical areas and used in developing countries. The CIAT 184 variety is used as a feed supplement for pigs, leading to good performance in the growing and finishing period [48]. In this case, the use of stylo could lead to problems in the diet of pigs due to its high fiber content, which could reduce feed intake and digestibility and, therefore, lead to reduced energy and nutrient supply, negatively impacting growth performance. The crude protein and lysine contents in stylo are lower than those in soybean meal, and all essential amino acids show the same pattern. However, its use remains interesting owing to its excellent resistance in low-fertility conditions [48].
- Finally, concentrates (pulp and juice) of other green plants such as grass and forage legumes) have been included in monogastric diets as protein feed. Stødkilde et al. [49] focused on the pulp and protein concentrate from white clover (*Trifolium repens* L.), red clover (*Trifolium pratense* L.), lucerne (*Medicago sativa* L.) and perennial ryegrass (*Lolium perenne*), outlining their potential use for monogastrics thanks to their high protein content, proper amino acid composition and high N digestibility.

#### 3.1.2. Oilseed By-Products 

Defatted by-products, such as meals, cakes, and expellers, are obtained from oil-bearing plant species in widespread cultivation for oil production. These by-products, such as those from linseed (*Linum usitatissimum*) and sesame (*Sesamum indicum*), remain rich in crude proteins and could be used as ingredients for animal nutrition. 

Rapeseed meal (RM) intended for animal feed is derived from the press cake that remains post oil extraction. Its protein content is about 35% DM, but with higher fiber content than SB (ADF ~19%, NDF ~26% DM) [18]. Rapeseed is high in its content of both sulfur-containing amino acids and phosphorus, even if its use in animal feeding is limited by the presence of antinutritional factors such as glucosinolates, tannins and phenols [50,51]. RM has been included in feeding mixtures for pigs, up to 15% of the total diet [51]. Recently, the canola meal (CM) from a variety of rapeseed was tested as a feed ingredient due to its low content of erucic acid and glucosinolate [52,53,54]. 

#### 3.1.3. Other Plants as Protein Sources

In recent years, other minor local plants have been tested as SB substitutes, including guar, sainfoin, taro, and others such as *Jatropha curcas* and *Moringa oleifera*. 

Guar (*Cyamopsis tetragonoba* L.) meal is a by-product of guar gum production. It contains highly viscous non-starch polysaccharides, such as galactomannan polysaccharide [55], which increases digesta viscosity, thus inhibiting digestive enzymatic activities and reducing nutrient digestibility [55]. 

Taro (*Colocasia esculenta* L.) is a perennial tropical plant native to Asia and the Pacific. The leaf of the taro plant is used as feed, as it has high protein content with a good amino acid profile. High levels of calcium oxalate crystals in the wild leaves are a cause of itching, but can be reduced by different processes such as drying, cooking, and ensiling [56].

*Jatropha curcus* is an Euphorbiacea cultivated in many tropical and subtropical regions. The kernel meal contains 48% to 64% crude protein after oil extraction, as reported by Li et al. [57]. However, in the above-mentioned study, many compounds toxic to animals were also identified, so this plant should be limited to non-toxic varieties or used only after specific treatments. 

#### 3.1.4. Algae

Algae are heterogeneous organisms that live in aquatic habitats and vary in size, including eukaryotic algae and prokaryotic cyanobacteria (blue algae). Depending on the size and structure of the body, algae can be classified into microalgae (observed under a microscope) or macroalgae (visible to the naked eye). Algae components can be used as natural supplements in human foods and animal feed in order to replace synthetic components [58]. A recent review reported that seaweeds can be a source of active compounds for pigs, such as polysaccharides, proteins and amino acids, and lipids including omega-3 and -6 fatty acids, vitamins, minerals and phenolic compounds [38,59,60,61].

Microalgae represent a source of protein and fiber for supplementation in human and livestock nutrition thanks to their high nutritive value, essential amino acid content, digestible protein, vitamins, minerals, carotenoids and fatty acids, especially long-chain omega-3 fatty acids [62]. Health-promoting effects characterize microalgae, such as antioxidant activity, immune stimulating properties and benefits for intestinal microorganisms [62]. Furthermore, microalgae have a rapid growth rate, and they can have a role in reducing the problem of soil availability, as they can derive from landless cultivation. Moreover, they have the peculiarity of being photosynthetic organisms that can transform carbon dioxide into carbohydrates, lipids, proteins and pigments [60]. Recently, microalgae have been used as feed supplements or additives in the swine sector [62,63,64]. 

Among microalgae, particular attention should be paid to spirulina (*Arthrospira platensis*), which can be used as a feed supplement immediately after drying. Spirulina is characterized by high crude protein content (43–63% DM) and small amounts of carbohydrates (8–14% DM) and lipids (4–9% DM) [7,65]. Spirulina consists of a unique blend of nutrients containing antioxidants, such as β-carotene, vitamin E and gamma linolenic acid. Moreover, *Spirulina* sp. offers the advantage of being cultivated in high saline water and alkaline conditions, making it an interesting feedstock for livestock [7,52].

#### 3.1.5. By-Products from Other Industries

Industrial processes, such as the extraction of starch from potato or other starchy vegetables, as well as the process for the production of bioethanol, can make available aqueous flows that include protein fractions.

**Figure 1:**
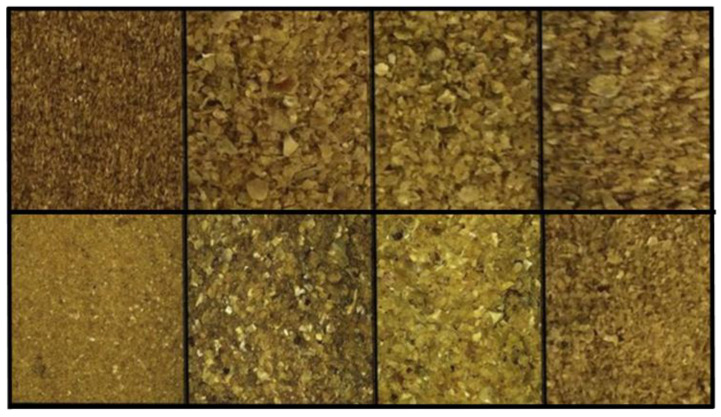
Corn distillers’ dried grain with solubles [68].

Distillers’ dried grain with solubles (DDGS) are by-products of the food industry that are considered a good source of protein and energy for animal feed. DDGSs are the main co-product from the ethanol industry produced by dry mill ethanol plants (Figure 1). These by-products are increasingly widespread due to the encouraged use of renewable energy sources for production of biofuels. Pecka-Kiełb et al. [66] reported a crude protein content of 25–30% DM for corn DDGS (cDDGS). In the past, it has been used mostly in ruminant diets considering the high fiber content, but recently it has also been used in monogastric diets thanks to its protein and fat content [67]. Recently, cDDGS was tested as a partial replacement for SB meal in pig finishing diets [9] thanks to its well-digested protein characteristics, low content of antinutritional substances and high nutritional values. However, as it is also rich in unsaturated fatty acids, it can negatively affect dietary intake, and consequently, the fatty acid profile of adipose tissue, and in particular the products’ oxidative stability [9].

Rice distillers’ by-product is also considered a good protein source, as it contains high values for crude protein. However, like many alternative protein sources, it is high in fiber, which can limit its use [45]. 

Food industry by-products also include supermarket and restaurant residues; in particular, those from bakeries and the production of snacks can be treated to become animal protein sources. This type of residue could represent good feed ingredients and, at the same time, reduce the amount of waste in the industries and processes involved [24]. Even if they can contribute to the replacement of SB, no research has tested their use in pig feed formulations.

### 3.2. Other (Non-Plant) Protein Resources

#### 3.2.1. Animals and Fish Proteins

The main processed animal proteins include meat and bonemeal, blood products, feather meal, and poultry by-product meals and are aimed at beef, swine, and poultry diet formulations [1]. However, in the past, due to the BSE outbreak in Europe, the use of animal proteins for feed formulations was forbidden. Nevertheless, processed animal proteins, if properly managed, can represent a valid protein source [24]. Mainly intended for young animals, liquid whey residues from the cheese industry are also produced and supplied as dried ingredients.

Innovative resources could be represented by products derived from industry or restaurants, supermarkets, or similar operations. Animal protein feeds can also include food industry by-products, for example, inedible meat, waste animal tissue and fat. 

Fishmeal by-products are traditionally used as an ingredient in animal feed when the fish does not reach the specified market size. For instance, Cândido et al. [69] tested the management of fish residues through the ensiling process, with controlled fermentation, to lengthen storage. The fish silage showed high protein content (39.01%), high protein digestibility (93.58%) [69], and high biological value, even if the high moisture content made its use difficult [22]. Additionally, due to the price and limited availability, its use has been reduced in poultry and swine diets, and it is estimated that its use will be further reduced in the future [24].

#### 3.2.2. Insect Meals

Insects have recently been considered a sustainable and promising high-quality protein source for feed formulations due to their high protein content and high fat content [70,71]. The presence of substances with beneficial effects on the health of pigs, such as chitin, lauric acid, and antimicrobial peptides, has been reported [72]. However, the cost of insect production is still considered too high to be competitive with traditional protein sources [70].

The larvae of the black soldier fly, *Hermetia illucens* L., convert organic resources (fruit residues, animal manure, vegetables, and cereals used by brewers) into high-quality proteins [71] containing about 40–44% crude protein with an amino acid profile comparable to that of SBM [53,73,74]. Furthermore, these larvae contain a variable percentage of fat from 7% to 39% [59,71] as well as calcium, phosphorus, sodium, and magnesium (Barragan-Fonseca et al., [73]). The research on their inclusion in pig feed has focused on piglets, replacing either fish meal or SBM [71,75,76], and on growing-finishing animals [77].

## 4. Effect of Alternative Protein Resources on Growth Performance and Slaughtering Traits

Several studies have investigated the results of SB replacement with other resources on in vivo performance and carcass traits. In this work, the main parameters taken into consideration were those related to growth, such as final body weight (BW), average daily gain (ADG), and feed conversion rate (FCR). In addition, other parameters, such as the average daily feed intake (ADFI) and dry matter intake (DMI), were also analyzed. Concerning carcass traits in addition to the yield and dimensional measurements, the composition in main commercial cuts and proportions of lean and fat were reported. 

A summary of research aimed at studying the effects of SB replacement on in vivo performance and carcass traits is reported in Table 1, including the feed sources, percentage of inclusion in pig diets, breed and weight of pigs, country and parameters analyzed. 

### 4.1. Plant Protein Resources 

#### 4.1.1. Legume Alternative to Soybean

Various studies tested the use of FB in swine feed as SB substitution ranging from 20 to 100% both on the in vivo performance and on the quality of the carcass. Milczarek and Osek [31], replacing about 28% of SBM with two varieties of FB in Pulawska pigs, found no effect on the main in vivo traits such as BW, ADG, and FCR. The same results were also obtained by totally replacing SB in the diet [31]. Smith et al. [41] observed no significant differences in ADG, ADFI, and FCR comparing a mixture with SB and various levels of included FB. The same results were also obtained regarding carcass parameters: both the measurements (i.e., carcass length, backfat thickness, loin eye area) and the percentages of the main tissues (meatiness, lard) were similar in the subjects given the two protein sources. In the latter case, Smith et al. [41] attributed the lack of differences to the addition of methionine and tryptophan to diets containing FB, considered deficient in these compounds [34]. Many studies, to cover the deficiencies that individual protein sources may have, have tested the total or partial replacement of soybean with mixtures containing FB combined with other vegetables. This was the case for Sobotka and Fiedorowicz-Szatkowska [34]. In their work, FB was tested with rapeseed meal (1:0.6 in the first period and 2:1 in the last period) replacing 50% of the soybean meal in the grower phase and 100% in the finisher phase. The final BW, ADG and FCR, as well as the main characteristics of the carcass, were the same for both tested periods. Sirtori et al. [32], using 22% FB as a total replacement for SB, did not record significant differences in the performance or carcass traits of outdoor-reared Cinta Senese pigs in the growing-finishing period, reporting the same dressing percentage, fattening state, and percentage of various tissues. Degola and Jonkus [29], replacing 100% of SB (15% of the whole diet) with 20 and 25% FB did not find differences in the fattening period, while they recorded a decrease in ADG in the growth period of the pigs. Regarding carcass traits, the same authors, with both inclusions, showed good results in carcass characteristics, though inclusion of 25% FB tended to yield less lean meat content, greater backfat thickness and more internal fat than the other groups. A complete replacement of SBM with an inadequate quantity (in %) of another legume as a substitute could cause a worsening of performance, failing to cover the needs of the animals. This problem is most evident in the early growth stages without a crystalline methionine or tryptophan supplement. Quander-Stoll et al. [78] reported a comparison between a conventional diet and a diet for use in organic farming, in which the addition of amino acids was not allowed and different percentages of FB and pea were used as substitutes for SB. The ADG of animals fed the conventional diet was higher than that of animals fed the organic diet, probably due to the lower lysine: energy ratio of the organic diet. In connection with this outcome, the authors also recorded a worse FCR in animals fed the organic diet. Lysine is the first limiting factor of protein synthesis and, consequently, of muscle growth. The methionine content was lower in the organic diet, and a methionine deficiency reduced growth performance with an increased fat deposition [78]. In this work, the same results were obtained for carcass traits, between the two diets, with small differences attributed to the dressing percentage. Some studies indicated that the inclusion levels of FB have to be lower than 20% in order to avoid decreased performance [36], even if there is no association between animal performance and tannin content. However, White et al. [36] indicated that grower and finisher pigs are able to tolerate a greater rate of FB inclusion (300 g/kg), provided the diets are nutritionally balanced using a variety of FBs with high tannin content. 

Regarding the use of pea, Smith et al. [41] proposed different levels of inclusion to substitute for SB, modifying the inclusion of crystalline AA. Compared to the diet containing SB meal, they reported similar results for in vivo performance, while noting fluctuating trends in the finishing phase for ADG. The authors did not explain this last result despite the fact that increasing the inclusion of legumes increases the intake of oligosaccharides, which often causes diarrhea in non-ruminants and, therefore, influences performance. In the case of pea and FB, their hulls may increase intestinal fluid absorption. However, Smith et al. [41], measuring fecal DM content and subjective visual fecal and cleanliness scores, showed that no loose feces or diarrhea occurred in pigs. Degola and Jonkus [29], replacing the total content of soybean meal with 15 and 28% of pea (in the growing and finishing periods, respectively), recorded no differences between the experimental results despite the lack of balance in methionine or tryptophan. However, despite the same performance compared to an SB diet, the authors noted an absolute value of ADG lower than in the previous tests in which crystalline methionine or tryptophan were added. White et al. [36], indicated that the use of legumes as potential alternatives to soybean meal should consider, irrespective of legume type, tannin content and sowing season. In the case of the total replacement of SB with 300 g/kg of pea, grower and finisher pigs were able to tolerate such inclusion if the diets were nutritionally balanced. In addition, Sirtori et al. [32] reported nutritional equivalence of pea and SB protein and the same performance with the two diets. 

In many recent papers, the partial or total replacement of SB was carried out with a mixture of different alternative protein sources. The inclusion of pea was always accompanied by other protein sources to balance the feed mixtures. Sońta et al. [79] reported good results for ADG and FCR, replacing total soybean meal with different levels of pea inclusion and rapeseed meal as supplement. Fiedorowicz-Szatkowska et al. [80] added 00-rapeseed meal (characterized by high concentrations of methionine + cystine and a ratio of lysine to methionine + cystine of 1:0.83) and pea in order to compensate for the lower biological value of pea protein compared to SB. Results showed that similar performance was achieved with both the pea and SB mixtures. However, increasing the use of alternative legumes as a protein source requires additional attention to components that can create problems with palatability, digestion, and low nutritional value. Zmudzińska et al. [18], testing two protein mixtures, pea and lupin in the growth period, and pea, lupin, and rapeseed in the finishing period, recorded decreasing performance. The first period was characterized by lower feed consumption, probably resulting from a lower tastiness of the alternative feed mix (including yellow lupin, pea and rapeseed), or due to the different post-prandial glycemic response of legume seed-based diets. Regarding the first case, the presence of antinutritional factors such as alkaloids or glucosinates (in lupin and rapeseed, respectively) can negatively affect performance [18]. Instead, the different physical and chemical characteristics of the polysaccharides can influence the digestion of the various feed sources [81]. Especially for monogastrics, the mechanism of digestion of complex carbohydrates is fundamental in terms of productive performance, digestibility, feed intake, quality of carcass and animal physiology. Some studies have shown that adding feed with enzymes may improve the digestibility. The use of glucanase, pectinase or hemicelullase can lead to the decomposition of polysaccharides in the seed cell walls. This increases the availability of nutrients for enzymes in the animal’s digestive tract [82]. 

Additionally, regarding the effects on the main carcass parameters, pea used in total or in partial replacement of SB has almost always yielded results comparable to the classic protein source. Izquierdo et al. [83], replacing 100% of soybean with various varieties of pea, did not observe significant differences in the lean and fat components of carcass. The same lack of differences was noted by De Quelen et al. [84], who tested two alternatives and more environmentally friendly diets that included P. De Quelen et al. [84], as well as Fiedorowicz-Szatkowska et al. [34], suggested comparable results in terms of carcass yield and lean components with respect to the SB group, even if the results could have been affected by different levels of growth performance. The results achieved by the authors were also attributed to the fact that they used a diet containing a variety of pea (*Albatros*) with a low content of tannins. Hanczakowska and Świątkiewicz [82], using a diet with pea substituting SB for 50% in the growing period and 100% in the finishing period, showed no differences in carcass fat meatiness and thickness of, as well as dressing yield and loin eye area. Degola and Jonkus [29] replacing SB with two levels of inclusion of pea, found no difference in carcass characteristics. Sońta et al. [12] and Smith et al. [41], gradually reducing the amount of SB used and increasing the percentage of pea administered, did not find differences in carcasses traits. 

Some works have foreseen the use of diets containing a mix of alternative protein sources, making the creation of correct rations more complex. Zmudzińska et al. [18], comparing a diet with SB with one of a mix of legumes (pea, lupin and rapeseed), reported a lower average backfat thickness with the alternative diet than with the soybean diet. This result is probably explained by the higher body weights at slaughter of SB-fed pigs. In addition, Quander-Stoll et al. [78], using a mix of pea and FB in an organic diet, reported a lower dressing percentage than that of the SB group due to the high crude fiber content in the organic diet resulting in high water-binding capacity and dietary bloating, which can cause more fiber-bound water in the intestines of animals and contribute to a lower dressing percentage. 

Lupin (*Lupinus* L.) represents another resource that seems to be useful overall if integrated with other crops as well as amino acid additions. This is confirmed by studies such as that of Degola and Jounkus [29], in which they reported a reduction in performance (ADG, ADFI and FCR) with the use of lupin alone as a substitute for SB compared to other legumes. Sobotka and Fiedorowicz-Szatkowska [80] reported the use of diets containing combinations of multiple protein sources precisely to overcome the limits of the individual feeds used. In their work, combining lupin with rapeseed, there was an increase in nutrient digestibility because it seems that the inclusion of lupin led to an improvement in protein and fiber digestibility. This led to an increase in performance with respect to the use of SB. However, the data of various works were conflicting. Stanek et al. (2004) found that increasing lupin content decreased crude fiber digestibility. Hanczakowska and Swiatkiewicz [82] found no differences in digestibility. Sobotka and Fiedorowicz-Szatkowska [34] reported no effect on carcass quality traits in grower and finisher diets including alternative vegetable protein sources such as lupin to partially or totally replace SB. The authors concluded that low-alkaloid lupin seeds can be a valuable high-protein feed ingredient alternative to soybean in pig nutrition.

In terms of carcass characteristics, most of the studies that compared the use of lupin with a traditional diet with SB reported no significant differences in the main parameters. Again, Hanczakowska and Swiatkiewicz [82], using two varieties of lupin, did not find any differences, albeit advising attention in the use of blue lupin due to the lower ingestion reported. Degola and Jounkus [29], with two inclusion levels of lupin, obtained good results for carcass traits also, without adding amino acid.

Inclusion of vetch (from 5 to 20%) was reported by Gómez-Izquierdo et al. [85], who noted a depression in daily feed intake and, therefore, a lower daily body weight gain up to 4 weeks after introduction of the novel feed, mainly for the 20% of inclusion. With the continuation of the trial, the pigs showed a light adaptation to the new diet, but less for the diet containing 20% vetch. The negative effect seems attributable to the bad taste of vetch. The same authors reported that the problem may be reduced with a longer transition period to the vetch diet and by genetic improvement of vetch to lower its antinutritional compound content. Phengsavanh and Lindberg [56], using another vetch type (*Aeschynomene histrix* BRA 9690), reported a decrease in performance in piglets between 15 and 50 kg with increases in the substitution of soybean (33; 66; 100%). The inclusion of vetch led to a lower DMI and hence, lower CP, amino acid and ME intake, which resulted in lower growth performance. The negative effect of this vetch was due to the increasing fiber content in the diet with increasing inclusions. The authors reported linear decreases in the main carcass traits with the increases in the inclusion of vetch, especially as regards the percentages and thicknesses of the fat component. The greatest differences were highlighted with the diet replacing 100% of SB. This was an effect of lower energy intake in pigs characterized by a high adipogenic capacity.

Among the minor alternative legumes, stylo (*Stylosanthes guianensis* Aubl., Sw. var. *guianensis*) is a legume that is native to South America. Kaensombath et al. [81] did not record negative effects on performance in pigs until 50% of replacement of soybean meal without the addition of crystal amino acids. However, the authors noted a reduction in the calculated dietary ME content of diets with increasing stylo inclusion, linked to an increase in DMI. Nevertheless, conflicting conclusions were shown: Phengsavanh and Lindberg [56] reported a negative effect with increasing inclusions of stylo as a substitute for SB. The authors reported a decrease in ADG and DMI linked to an increase in FCR. All of these seem to be linked to the higher fiber content of the stylo, which led to a reduction in the DMI and, consequently, in the protein intake. The low available digestible energy was caused by higher NDF content and lower hemicellulose, cellulose and lignin fractions not supplying enough highly fermentable hemicellulose [47]. 

Regarding the effect on carcass traits, it seems that the influence of the protein source depends on the degree of substitution of the soybean by stylo. No significant difference was noted from an SB diet up to about 50% substitution, but at higher percentages of substitution, the effects were evident, especially for fat tissue [48,56].

#### 4.1.2. Oilseed By-Products

In the studied period, research on oilseed by-products for pig feed was mainly focused on rapeseed meal (RM). Works in the literature reported that RM was integrated at the same level as an alternative protein source, such as legume seeds [80,82], while some trials used it as the main source for replacing SB. The addition of RM involved a decrease in feed intake due to the presence of glucosinolate, which reduced the tastiness of the diet [18,34]. Sobotka and Fiedorowicz-Szatkowska [34] tested three diets with different inclusions of 00-rapeseed meal in the growing period (50% substitution with only rapeseed meal; 50% substitution with RM and FB mixture; 50% substitution with rapeseed and lupin mixture) and a total replacement in the finishing period with respect to the control diet with soybean meal. They found no significant differences regarding ADG and FCR, demonstrating the flexibility of using this protein source alone or together with other resources. Further, Hanczakowsk and Świątkiewicz [82] concluded that rapeseed can be a good addition to other legumes to replace soybean without having negative results. Skoufos et al. [86] reported that in order to replace 100% of soybean meal with rapeseed meal, it is necessary to add synthetic amino acids to the mixture to balance the nutritional quality. In this way, they managed to have no differences in performance compared to the use of soybean and with respect to other works in which the addition was not present. However, the results were contrasting. The inclusion of RM can exceed 20% without compromising growth performance, whereas others have shown that with rapeseed meal inclusion, a percentage of less than 20% can compromise some performance parameters. These contradictory results may arise from differences in the glucosinolate concentrations due to the use of different varieties of rapeseed. The negative impact of rapeseed meal found, for example, by Torres-Pitarch et al. [87], on the growth performance of pigs, could be linked precisely to the high amount of anti-nutritional factors present in the variety used. 

The inclusion of RM as total or partial substitution of SB did not seem to influence the characteristics of the carcass. Sobotka and Fiedorowicz-Szatkowska [34] reported no difference in carcass traits (dressing percentage, backfat thickness, lean content) with inclusion of rapeseed both as a total substitute of soybean and in mixtures containing multiple alternative protein sources. The inclusion of RM also as partial substitution of SB had no significant effect on the main carcass traits such as weight, carcass yield, carcass lean percentage, composition of cuts, and fat thickness [87]. In most cases, the use of RM as an addition to diets with other main protein sources did not show effects on the carcass [82] except for slight variations in values related to fattening [18,80]. 

#### 4.1.3. Other Plants as Protein Sources 

In recent years, other minor local plant sources have been tested as substitutes for soybean, including guar and sainfoin or others such as *Jatropha curcas* and *Moringa oleifera*. The use of these sources is linked to feed efficiency. Various factors, including nutrient digestibility and availability, palatability and animal adaptation to new feed ingredients, have an impact on feed intake and thus on performance. For this reason, the results can be inconsistent with each other based on the length of the trial period, which can allow more or less wide adaptation, and on the amount of antinutritional factors [55,57,88].

Guar meal inclusion in the diets of Yorkshire × Landrace pigs resulted in a decrease in average daily feed intake [55]. This affected the other parameters, such as ADG and FCR, which presented a linear decrease with increases in the inclusion of guar. The problem is linked to the unpalatability of guar. No recent research has examined the effects of guar inclusion on carcass traits.

Sainfoin bean was tested by Seoni et al. [88] up to a 15% inclusion (about 40% soybean replaced), together with 12.0 g/kg DM of condensed tannins. Animals showed growth rates comparable to those of the control group. However, the authors noted behavioral differences with increased sainfoin inclusion, recording longer feeding times with fewer visits to the feeder. The diets were isonitrogenous and isocaloric; only the tannin, crude fiber and fat content differed. For this reason, the authors linked the altered feeding behavior pattern to increased fiber-induced satiety; indeed, the amount of ingested dietary fiber increased on average by 2.6, 8.1, and 10.7 g/kg. The authors suggested that the quantity was too low to reach bulking properties that impact meal size. The dietary fat content linearly increased from 7.7 to 23.7 g/kg of feed, and no evidence was found that feeding behavior was affected by this factor, especially at the moderate levels used in their trial. The tannin content of the diet did not affect overall feed intake; for this reason, it was hypothesized that the present astringency was not sufficient to markedly affect the total feed intake, but it nevertheless led to a slowdown in eating speed. The authors reported, in conclusion, that a 7% inclusion can be used in the diet without having negative impacts on performance. Regarding carcass parameters, Seoni et al. [88] reported only differences in carcass yield, while the results for other traits were comparable to the control diet with soybean.

Taro leaves were used by Kaensombath and Lindberg [48] to replace up to 50% of soybean protein in the diet of growing pigs without negative effects on performance and carcass traits. This is thanks to the protein content and the lysine–energy ratio present in the diet with taro, which made it possible to have no growth deficiencies in the trial. However, the authors recorded a reduction in ME content from −3.8 to −9.2, at different stages of growth, with the increased inclusion of taro leaf silage, which did not adversely affect performance due to the balance created by the increase in DMI. Taro leaves did not lead to significant differences in the quality of the carcass up to a replacement of 50% of the soybean [48].

Moringa oleifera was studied by Rivero et al. [89], who tested its inclusion in the swine diet instead of soybean up to 40%. The gradual increase in Moringa resulted in decreased performance both for ADG (from 602 to 473 g/d) and for average daily feed intake (ADFI) (2.04 to 1.48 kg/d) despite the slight improvement in FCR. The authors attributed the results to the higher percentage of fiber in Moringa compared to corn and soybean cake. 

*Jatropha curcas*, as detoxified kernel meal, was used in a trial by Li et al. [57]. With their procedure, the authors tried to replace up to 75% of the soybean present in the diet. Despite the excellent protein content and amino acid profile, the authors reported that pigs fed with less than 30% replacement of soybean with Jatropha showed good ADFI, ADG and FCR, but, when the replacement of SB with this source was higher than 30%, adverse effects were observed in ADFI, ADG and G:F. The results obtained could be attributed to the palatability and the presence of toxic and anti-nutritional factors. The negative results in ADFI could be attributed to the taste, smell, and texture, but above all, to the excess of phorbol esters in these diets. The lower digestibility and absorption of nutrients could be the reason for the reduced growth performance. The present study demonstrated that substitution of soybean by greater than 30% resulted in a dietary phorbol ester concentration of 5.50 mg/kg. This could have decreased the use of energy and nitrogen and the enzymatic activities, damaging the intestinal morphology. Li et al. [57] reported only limited intestinal development and jejunal villus structural changes, such as disturbances and ruptures, with more than 30% soybean substitution. Poor digestion and absorption would seriously affect the growth and health of the animal.

#### 4.1.4. Microalgae

Spirulina (*Arthrospira platensis*), a microalgae, was tested by Altmann et al. [7] in a feed trial on Pietrain × (Large White × Landrace) as replacement for 50 or 75% of soybean. The study observed the same slaughter weights at the end of the trial, taking into account that the aim was to test the use of seaweed on the quality of the product. Other works tested the use of spirulina but only as a small addition, such as that by Šimkus et al. [90], which found improvements in ADG performance and faster achievement of slaughter weight.

Martins et al. [91], in their work on weaned piglets, studied the replacement of 5% of soybean with Chlorella vulgaris with or without the use of enzymes. This inclusion did not compromise the productive variables, unlike Martins et al. [92] in a previous article, which reported that a 10% replacement led to reduced growth performance. Despite the greater use of microalgae as additives in diets and not as the main protein source, their use as a substitute for high percentages of SB has shown excellent results with regard to the effects on the carcass. Altmann et al. [7] found no differences in carcass parameters (i.e., lean meat yield) in their soybean replacement trial with spirulina.

#### 4.1.5. By-Products from Other Industries

Other sources used as soybean substitutes are rice by-products such as defatted rice bran and rice distiller’s grain. Huang et al. [93] reported that defatted rice bran can be a good solution to partially replace soybean use, highlighting any negative effects on performance with different levels of inclusion, from 2.5 to 20%, in different stages of growth. Casas et al. [94] conversely reported negative effects on performance with an increase in feed intake and decrease in ADG and FCR. This latter result was probably due to higher (inclusion) percentages of defatted rice bran up to 30%, together with higher fiber content, which usually leads to satiety and causes animals to stop eating earlier. On the other hand, they found no differences in the quality of the carcass, including with full fat rice or defatted rice in the diet.

#### 4.1.6. Insects

Several species of insects have been considered as a sustainable alternative to meet the demand for partial or complete replacement of conventional protein feeds such as soybean [77]. Among the insects most considered as animal feed are the black soldier fly (*Hermetia illucens* L.) and yellow mealworm (*Tenebrio molitor* L.) [95]. Insects as a feed source seem to be a good raw material, both in the growing and finishing phases. Biasato et al. [76], in a work with piglets (20 days of age), tested diets prepared to include, on a feed basis, increasing levels of black soldier fly larva meal in substitution of 0%, 30% and 60% of soybean meal from 1 to 23 days of the trial, or 0%, 32% and 65% from 24 to 61 days. The inclusion of black soldier fly larva meal in the diet did not influence the piglets’ growth performance, although a minimal increase in ADFI was noted. This could be attributed to a greater palatability of the diet, given by the presence of the insect. Yu et al. [77] conducted a study to evaluate the effects of dietary inclusion of *H. illucens* larva meal in the diet of finishing pigs, replacing soybean meal (from about 18 to 36%). The authors recorded significant changes only with the diet with the lower inclusion of insects, in particular an increase in ADG and a decrease in FCR. The lack of effect from the diet with the higher content in *H. illucens,* compared to the soybean diet, could be due to the higher level of chitin in the diet, which did not allow for improvement. With regard to ADFI, the two diets both proved to be acceptable for finishing pigs. Additionally, Meyer et al. [96], using *Tenebrio molitor* L. as substitute for 50 or 100% of soybean, did not find significant differences in in vivo performance except for a decrease in ADG with inclusion of the insects, although the authors attributed part of the difference to the unequal stocking density in each treatment group.

Considering carcass traits, in the work by Yu et al. [77], the diets with *H. illucens* did not affect the main parameters (carcass weight, carcass yield, average backfat) with the exception of the area of the loin, which was greater in the diet with 18% replacement of soybean.

#### 4.1.7. Sources of Animal Origin

Other sources used to replace soybean include the residues and by-products of food processing aimed at human nutrition. In this context, residues from fishing or from chicken farming stand out. Candido et al. [69] tested three percentages of inclusion (25–50–75%) of fishmeal as a soybean substitute, varying within three stages of pig growth due to the decrease in the percentage of soybean administered in the three phases: phase 1, from 30 to 50 kg of body weight, with 10–18–26% substitution of soybean; phase 2, from 50 to 70 kg, with 10–19–30% substitution; phase 3, from 70 to 100 kg, with 10–22–34% substitution. The work highlighted good results with the lowest substitution of soybean, showing decreasing performance (daily feed intake, daily weight gain and feed conversion) with the higher percentages, especially with the highest substitution tested. Similar results were found in the qualitative traits of the carcass. A higher inclusion showed a decrease in some parameters such as back fat thickness, loin depth, and fat depth. Trabue et al. [97], on the other hand, by replacing 100% of soybean with poultry meal did not show significant differences with the basic diet in terms of performance (ADFI, ADG and FCR).

**Table 1:** Summary of studies aimed at assessing the effect of soybean replacement on growth performance and carcass traits.

Item	SB Replacement (%)	BW	ADG	ADFI	FCR	Dressing Percentage	Meatiness	Carcass Measurements	Backfat Thickness	Breed	Country	Initial–Final Weight	Authors
Moringa oleifera	50; 100	X	X	X	X	X	X			Duroc × Yorkshire × Landrace	Cuba	20–65	Rivero et al. [89]
Rapeseed meal	38; 62	X	X	X	X					Pietrain × (Landrace × Large White)	Spain		Torres-Pitarch [87]
Legumes	-	X	X	X	X	X	X	X	X	Large White × Duroc	Congo	25–75	Kambashi [47]
Vetch, stylo	34; 60	X	X	X	X	X	X	X	X	Moo Lath	Lao	14–50	Phengsavanh and Lindberg [56]
Pea, fava	100	X	X	X	X					Pietrain × (Large White × Landrace)	France	40–110	De Quelen [84]
Pea	100	X	X	X	X		X		X	Duroc	Spain	22–120	Izquierdo et al. [85]
Tenebrio molitor	50–100	X	X	X	X		—				Germany	Piétrain × (German Landrace × German Edelschwein)	Meyer et al. [96]
Hermetia illucens	Different percentages	X	X	X	X					commercial piglets	Italy	5–33	Biasato et al. [76]
Insect meal										review	Australia		DiGiacomo [95]
Fat rice bran and defatted rice bran	Different percentages	X	X	X	X	X	X		X		USA	28–120	Casas et al. [94]
Spirulina		X	X	X	X					Landrace × Yorkshire	Lithuania	30–100	Šimkus et al. [90]
Pea, fava bean	25; 50; 75			X	X	X	X		X	Large White × Landrace	England	45–100 kg	Smith et al. [41]
Defatted, rice bran	Different percentages	X	X	X	X					Duroc × Landrace Yorkshire	China	25–100	Huang et al. [93]
Jatropha curcas	15; 30; 45; 60; 75	X	X	X	X					Duroc × Landrace × Yorkshire	China	20–75 kg	Li et al. [57]
Guar	25; 50; 75; 100	X	X	X	X					Yorkshire × Landrace	USA	30–65 kg	Hasan et al. [55]
Pea, fava bean	100	X	X	X	X	X		X		Cinta Senese	Italy	45–135 kg	Sirtori et al. [32]
Fava bean cv albus or amulet	28	X	X	X	X	X	X	X	X	Puławska	Poland	31–117 kg	Milczarek et al. [31]
Fava bean, rapeseed, lupin	50 to 100	X				X	X		X	Hybrid Danbred	Poland	26–104 kg	Sobotka et al. [34]
Pea, fava, lupin	pea 15–28; fava 20–25; lupin 12–15	X	X	X	X	X	X	X	X	Yorkshire × Landrace	Latvia	30–100 kg	Degola and Jonkus [29]
Fava + pea	100	X	X	X	X	X	X	X	X	F2-piglets	Switzerland	106 kg	Quander-Stoll et al. [78]
Pea or fava	100	X	X	X	X					commercial white hybrid	England	30–95 kg	White et al. [36]
Pea seed, rapeseed meal	Stage 1: RSM + P 2.5 + 5.0; 2.5 + 10; 2.5 + 15; 7.8 + 17.5; Stage 2: RSM + P 2.5 + 5.0; 2.5 + 10; 2.2 + 15; 6.0 + 17.5;	X	X		X		X			Danish porkers	Poland	27–125 kg	Sońta et al. [79]
Sainfoin	5, 10, 15	X	X	X	X	X	X	X		Large White	Switzerland	25–105 kg	Seoni et al. [88]
Taro leaf	25–50	X	X	X	X	X	X	X	X	Landrace × Yorkshire	Lao	11–45	Kaensombath and Lindberg [48]
Rapeseed meal	100	X			X			X		(Large white × Landrace) × Duroc	Greece	-	Skoufos et al. [86]
Rapeseed cake + (field bean/pea/blue lupin/yellow lupin)	43	X	X	X	X	X	X	X	X	Polish Landrace × (Duroc × Pietrain)	Poland	30–114 kg	Hanczakowska and Świątkiewicz [82]
Low-tannin pea cv. Albatros, rapeseed meal/high-tannin Fava bean + rapeseed meal	100	X	X	X	X	X	X	X	X	(Polish Large White × Polish Landrace) × Duroc	Poland	65–105 kg	Fiedorowicz-Szatkowska et al. [80]
Rapeseed, pea, yellow lupin	100	X	X	X	X		X	X	X	commercial hybrid	Poland	30–120 kg	Zmudzińska et al. [18]
Chlorella vulgaris	20	X	X	X	X					(Large White × Landrace) × Pietrain	Portugal	11–23 kg	Martins et al. [91]
Spirulina, hermetia illucens	50	X					X			(Landrace × Large White) × Pietrain	Germany	22–110 kg	Altmann et al. [7]
Hermetia illucens	18, 36	X	X	X	X	X		X		Duroc × Landrace × Large White	China	76–115 kg	Yu et al. [77]
Fish meal sileage	10, 22, 34	X	X	X	X	X	X		X	-	Brazil	26–100 kg	Candido et al. [69]

## 5. Effects of Alternative Protein Sources on Physical and Chemical Quality Traits of Meat

Concerning meat quality, several studies have investigated the consequences of SB replacement on the main characteristics of meat. The present review focused on discussing pH, color, texture and water-holding capacity among the main physical traits, and moisture, protein, fat and ash, fatty acids (FA) profile and TBARS contents among the main chemical traits. Furthermore, a few studies have also investigated meat sensory traits, as reported in Section 6. A summary of the parameters studied and of the impacts of SB replacement on meat quality characteristics are reported in Table 2 and Table 3, respectively. 

### 5.1. Replacement with Plant Sources

#### 5.1.1. Legumes

FB and pea were the legumes most used as alternatives to SB. However, their effects on meat quality traits were often contrasting. Milczarek et al. [31] tested the replacement of 28% SB with two varieties of low-tannin FB. Among the studied traits, only drip loss of meat from pigs fed the Albus FB variety was reduced if compared with the SB-fed group. Additionally, the Amulet FB variety increased the content of PUFA in meat when compared with the SB group. The other traits examined (pH, color, chemical composition) were unaffected by SB replacement. Similarly, pH, color, water-holding capacity, and tenderness were not affected by replacing 42% of SB with pea, or 58% with FB [33]. Instead, totally replacing SB with FB or pea brought some differences in color, as observed by Sirtori et al. [32], who reported an increased a* value in the group fed FB. As regards chemical composition, meat from pigs fed the diet with pea and FB was lower in moisture, whereas crude protein was higher in the pea diet and lower in the FB diet [33]. Sirtori et al. [32] observed modifications in meat chemical composition only in the group fed with pea, resulting in higher moisture and fat content compared with the SB-fed group. 

Different levels of SB replacement with alternative legumes were tested by Rauw et al. [98] and Seoni et al. [88]. The former investigated the effects of increasing replacement levels of SB with Narbon vetch (NV) on meat chemical composition, observing that replacing SB by up to 20% did not affect the moisture, fat and protein content of the meat. Contrarily, at increasing levels of SB replacement with sainfoin [88], a significant linear increase in pH at 3 h post-mortem as well as of drip loss were observed. A tendency of hardness to linearly increase was also observed. Eventually, color remained unaffected by the SB replacement levels. Several changes in the FA profile were also observed, pinpointing a linear decreasing trend in SFA, whereas MUFA quadratically increased as the percentage of SB replacement was raised [88]. Total PUFA was unaffected by dietary modification, as C18:2n-6 levels did not vary with increasing SB replacement levels. However, C20:2n-6 and C22:4n-6 decreased, while C18:3n-3 and C22:5n-3 levels linearly increased accordingly with higher percentages of sainfoin. This led to a significant linear decrease in the ∑n-6-to-∑n-3 fatty acid ratio, as well as to an increase in the desaturation index (defined as the C18:1n-9/C18:0) of meat.

#### 5.1.2. Oilseed By-Products

Only two studies reported the effects on meat quality after replacing SB with oilseed by-products. Little et al. [54] testing 33, 66 or 100% SB replacement with canola meal (CM) from two different varieties (conventional or high-protein). None of the investigated parameters (pH, color, texture, WHC, and chemical composition) were affected by SB replacement except for L*, which linearly decreased as the level of high-protein CM increased. Total replacement of SB using RM was also investigated [86]. In this case, the dietary treatment had a great impact on the chemical composition of different pork cuts; it decreased the fat content of shoulder and steak, while increasing the same parameter in ham and belly. Additionally, crude protein and moisture varied accordingly with fat content. Concerning the FA profile, the RM-fed group showed lower contents of SFA and PUFA in steak, whereas MUFA increased [86]. At the level of single FAs, SB replacement by RM affected C16:1, C17:0, C18:1n-9c, C18:2n-6c, and C18:3n-3 contents in steak, as well as C18:3n-3 and C20:2 contents in shoulder. Skoufos et al. [86] also investigated the lipid oxidation of ham and steak at 4 and 7 days, observing that SB replacement with RM had no effect on pork stability during storage.

Eventually, six studies investigated the use of different combinations of alternative protein sources to obtain an optimal SB replacement in terms of nutritive value and FA profile of the diet. Total replacement of SB was generally performed in the finisher diet. Experimental dietary treatments tested by Hanczakowska and Swiatkiewicz [82], consisting of different legumes (pea, field bean, blue lupin, or yellow lupin) in combination with rapeseed press cake, did not affect WHC and color. Instead, Fiedorowicz-Szatkowska [80] observed that the combination of low-tannin pea and RM, or high-tannin FB with RM, as SB substitutes did not affect meat chemical composition and PUFA, but increased MUFA contents. SFA was lower in the pea + RM group than in the SB group, while in the FB + RM fed group, it was similar to both. The research of Zmudzińska [18] confirmed the results of the previous studies; indeed, the SB replacement in the fattener diet with a combination of RM, pea, and yellow lupin did not affect either meat chemical composition or physical traits (pH, color, and WHC). Partially in agreement with that finding, Sońta [79], studying different levels (37, 52, 68, 100%) of SB replacement, concluded that the experimental diets did not affect meat physical traits (color and WHC), but slightly increased meat protein content. Moreover, in agreement with results reported by Fiedorowicz-Szatkowska [80], increasing levels of SB replacement, up to total substitution, reduced the SFA content in meat. However, Sońta [79] also observed an increase in PUFA with increasing levels of SB replacement, whereas MUFA was unchanged.

#### 5.1.3. Other Plant Protein Sources

One study examined the effects on meat quality traits of using different vegetable resources as SB substitutes. Using sunflower meal, FB, and potato protein as total replacement of SB in a fattening diet, Mordenti [99] did not observe any modification in pH, color and chemical composition of meat from animals fed the experimental diet. 

#### 5.1.4. Microalgae

Among the most innovative feeds, microalgae have been gaining attention in the last few years thanks to their high protein content and the potential to cultivate them without using arable land [7]. Total replacement of SB with spirulina in a finisher diet was tested by Altmann [7]. Concerning meat quality, none of the examined traits were affected by SB replacement with spirulina. Instead, Spirulina was tested as partial (56%) replacement of SB as protein source [91]. Meat quality traits were assessed on samples from piglets slaughtered 28 days after weaning, and some modifications were observed on TBARS, which was significantly higher in the samples at day 3 after slaughter. In line with this finding, the FA profile was slightly modified by spirulina dietary inclusion due to its high PUFA content. Increased content of C18:3n-6 was observed in the meat. The same authors also tested the use of Chlorella vulgaris to replace 20% of SB during post-weaning [91]. Examining the meat quality traits, they observed minor modifications in meat quality, likely due to the smaller extent of SB replacement. Cooking loss was reduced in meat from animals fed Chlorella vulgaris, but TBARS was slightly increased at day 8 in meat from microalgae-fed animals, likely due to the higher contents of PUFA than in the SB-fed group.

#### 5.1.5. By-Products from Other Industries

The use of corn distiller’s dried grain with solubles (cDDGS) to replace 50% of SB in the diet was investigated [9]. Since cDDGS is characterized by high content of unsaturated fatty acids, it can negatively impact meat quality and technological traits. For these reasons, the authors investigated the use of coconut oil or beef tallow together with cDDGS to cope with the increased risk of oxidation or changes in texture. As expected by the authors, SB partial replacement with cDDGS, without addition of beef tallow or coconut oil, led to a decrease in SFA in favor of unsaturated FA (MUFA + PUFA). Contrarily, the use of beef tallow in addition to cDDGS maintained the SFA and unsaturated FA contents similar to their levels in meat from the SB group. Coconut oil addition to cDDGS further raised SFA and decreased unsaturated FA content in meat compared with the SB group. Furthermore, experimental diets containing cDDGS did not affect the pH, color, and lipid oxidation of meat. The use of cDDGS combined with beef tallow or coconut oil had no significant impact on meat hardness, but positively affected WHC, which was greater for all groups fed cDDGS compared with the SB group. Further, the chemical composition of the meat was affected by SB replacement, which decreased the protein and increased the fat content.

### 5.2. Replacement with Sources of Animal Origin

#### 5.2.1. By-Products of Animal Origin

In the period considered by the present review, only one study evaluated the use of animal by-products to replace SB [91]. In this specific case, the authors tested three different levels of fish meal silage inclusion to replace 10, 22, or 34% of SB. None of the tested levels resulted in any modification of the physical and chemical quality traits studied.

#### 5.2.2. Insect Meal

Among the novel ingredients in feed, the use of insect meal is becoming more widespread. However, very few studies have investigated the effect of using insect meal for pork. The inclusion of *Hermetia illucens* meal for replacement of 40 and 30% of soybean meal significantly increased meat marbling [77]. In addition, the use of insect meal significantly affected some single FAs, but not the totals of SFA, MUFA, or PUFA. In details, the inclusion of 4% *Hermetia illucens* in the diet led to increases in C18:3, C20:4, C20:5, and C22:6 and the sum of n-3 polyunsaturated FAs compared with the control diet. Furthermore, the inclusion of 8% insect meal significantly increased the concentrations of C12:0, C14:0, C16:1, C20:4, C20:5, and C22:6 compared with the control diet, but decreased the concentration of C18:3 [77]. Contrarily, total replacement of SB with *Hermetia illucens* during finishing did not modify the examined meat quality traits (pH, instrumental tenderness, color, water content, cooking loss, TBARS, or chemical composition) [7].

## 6. Effects of Alternative Protein Sources on Sensory Quality Traits of Meat

Meat color, marbling, and firmness were evaluated by Little [54] in order to evaluate the feasibility of replacing soybean with increasing levels of canola meal. The authors did not observe any difference in the examined traits. The sensory evaluation of meat from pigs fed two different varieties of fava bean as partial replacement for SB underlined several modifications in the studied traits [31]. Specifically, tenderness, juiciness and palatability scored lower for meat of the soybean group than the fava bean-fed groups, whereas flavor was not affected. The inclusion of yellow lupin in the diet to replace soybean meal resulted in poorer scores for odor and taste, whereas juiciness was negatively affected by the inclusion of blue lupin [82]. Conversely, replacing half of the dietary SB with cDDGS was observed to enhance the tenderness and juiciness of meat [9].

The use of fish meal silage as partial soybean replacement affected the hardness and overall acceptability of meat, which linearly decreased as the fish meal silage in the diet increased [91]. Three (overall odor, astringent aftertaste, juiciness) of the 26 sensory attribute studied by Altmann [7] were found to be affected by diet. Indeed, both spirulina and *Hermetia illucens* protein sources resulted in stronger overall odors compared to the meat from the control group. Moreover, *Hermetia illucens* resulted in significantly higher juiciness values compared to the control and spirulina groups. According to the authors, this could be linked to the lower cooking losses as well as the high IMF values and could have favorable outcomes in terms of consumer acceptance. Conversely, spirulina showed the highest scores for astringent aftertaste, which could negatively affect eating quality. Spirulina was tested both as partial replacement of SB and as total protein source [91]. In addition to meat quality traits assessed in meat from piglets slaughtered 28 days after weaning, the study also evaluated the sensory profile of meat. Panelists reported a greater score for the flavor of meat from animals fed spirulina than that from control animals. However, the flavor was not affected by 20% SB replacement with *Chlorella vulgaris* [91].

## 7. Conclusions

In pig farming, soybean is traditionally the main source of feed protein, but in recent years this ingredient has increasingly become a limiting factor due to rising prices, ethical issues, environmental impact, and competition for feed/food for land use.

The dry seeds of legumes, especially if used as a combination of species, remain the most accessible protein resources as substitutes for soybean and, if balanced within the diet, they do not seem to affect performance and carcass/meat quality traits. Local species of legumes currently represent the only alternative with the potential of totally replacing SB. Among other potential substitutes, insect meal, micro and macroalgae and other products originating from aquaculture are of increasing interest. However, their use needs further research in the swine sector to better assess both their cost-effectiveness and their effects on meat chemical (e.g., fatty acid profile) and technological properties (e.g., iodine number). Specific processing techniques, protein sources for feed and alternative by-products, for example, those of other sectors, could represent an opportunity in terms of both costs and sustainability but also of valorization and recycling of products.

However, nutrient variability, digestibility and antinutritional effects should be evaluated when new ingredients are introduced into pig diets.

Further research should entail the combination of ecofriendly, sustainable, and local feed in pig diets, to increase the rate of soybean replacement without interfering with growth performance and the quality of the carcasses and the meat. Finally, specific effects on animal health and welfare and feeding behavior are recommended as targets for future research.

## Figures and Tables

**Table 2:** Summary of studies aimed at assessing the effects of soybean replacement on meat quality traits.

Item	SB Replacement (%)	pH	Color	Texture	WHC	Chemical Composition	TBARs	FA Profile	Sensory	Breed	Country	Initial–Final Weight	Authors
Pea, fava bean	42 pea; 58% fava bean	X		X	X	X				Pic × Goland	Italy	56–160 kg	Gatta et al. [33]
Pea, fava bean	100	X	X	X	X	X		X		Cinta Senese	Italy	45–135 kg	Sirtori et al. [32]
Fava bean cv albus or amulet	28	X	X		X	X		X	X	Puławska	Poland	31–117 kg	Milczarek et al. [31]
Sainfoin	5; 10; 15	X	X	X	X	IMF		X		Large White	Switzerland	25–105 kg	Seoni et al., [88]
Narbon vetch	5; 10; 20					X			X	Duroc × Iberian	Spain	60–174 kg	Rauw et al., [98]
Canola meal (convention or high protein)	33; 66; 100	X	X	X	X	X			X	Commercial hybrid	USA	57–114 kg	Little et al. [54]
Rapeseed meal	100					X	X	X		(large white × landrace) × duroc	Greece	-	Skoufos et al. [86]
Rapeseed cake + (field bean/pea/blue lupin/yellow lupin)	100		X		X				X	Polish Landrace × (Duroc × Pietrain)	Poland	30–114 kg	Hanczakowska and Świątkiewicz. [82]
Low-tannin pea cv. Albatros, rapeseed meal/high-tannin fava bean + rapeseed meal	100					X		X		(Polish Large White × Polish Landrace) × Duroc	Poland	65–105 kg	Fiedorowicz-Szatkowska et al. [80]
Rapeseed meal + pea, yellow lupin	100	X	X		X	X				commercial hybrid	Poland	30–120 kg	Zmudzińska et al. [18]
Rapeseed meal, pea	37, 52, 68, 100		X		X	X		X		(Landrace × Yorkshire) × duroc	Poland	26–123 kg	Sońta et al. [79]
Corn distiller’s dried grain with solubles (rapeseed oil/beef tallow/coconut oil)	50	X	X	X	X	X	X	X	X	(Polish Landrace × White Large Polish) × (Duroc × Pietrain)	Poland	60–118 kg	Hanczakowska and Świątkiewicz [82]
Sunflower meal, fava bean, potato protein	100	X	X		X	X				Landrace × Large White	Italy	50–160 kg	Mordenti et al. [99]
Spirulina	56	X	X	X	X	IMF	X	X	X	(Landrace × Large White) × Pietrain	Portugal	12–28 kg	Martins et al. [91]
Chlorella vulgaris	20	X	X	X	X	IMF	X	X	X	(Large White × Landrace) × Pietrain	Portugal	11–23 kg	Martins et al. [91]
Spirulina, hermetia illucens	100	X	X	X	X	X	X		X	(Landrace × Large White) × Pietrain	Germany	22–110 kg	Altmann et al. [7]
Hermetia illucens	18, 36	X	X	X	X	X		X		Duroc × Landrace × Large White	China	76–115 kg	Yu et al. [77]
Fish meal sileage	10; 22; 34	X	X	X	X				X	-	Brazil	26–100 kg	Candido et al. [69]

**Table 3:** Effects of using alternative protein sources on the studied meat quality traits as compared to soybean-fed control groups.

Item	SB Replacement (%)	pH	Color	Hardness ^4^	WHC ^5^	Chemical Composition	FA Profile	TBARs	Authors
Pea	42	ns ^1^	- ^2^	ns	ns	M ↑ ^3^ P ↑	-	-	Gatta et al. [33]
Fava bean	58	ns	-	ns	ns	M ↑ P ↑	-	-	
Fava bean	100	ns	A * ↑	ns	ns	ns	-	-	Sirtori et al. [32]
Pea	100	ns	ns	ns	ns	M ↑ F ↑	-	-	
Fava bean cv. albus	28	ns	ns	-	↓	ns	ns	-	Milczarek et al. [31]
Fava bean cv. amulet	28	ns	ns	-	ns	ns	↑ PUFA	-	
Sainfoin	13	↑	ns	↑	↑ (drip loss); ns (cooking loss)	ns (IMF)	SFA ↓ MUFA ↑ n6/n3 ↓ SFA/PUFA ↓	-	Seoni et al. [88]
	27								
	41								
Narbon vetch	5	-	-	-	-	ns	-	-	Rauw et al. [98]
	10	-	-	-	-	ns	-	-	
	20	-	-	-	-	ns	-	-	
Canola meal, conventional	33; 66; 100	ns	ns	ns	ns	ns	-	-	Little et al. [54]
Canola meal, high protein	33; 66; 100	ns	L * ↓	ns	ns	ns	-	-	
Rapeseed meal	100	-	-	-	-	M↑ P ↑ F ↓	SFA ↓ PUFA ↓ MUFA ↑	ns	Skoufos et al. [86]
Rapeseed cake (field bean/pea/blue lupin/yellow lupin)	100	-	ns	-	ns	-	-	-	Hanczakowska and Świątkiewicz [82]
Low-tannin pea cv. Albatros, rapeseed meal	100	-	-	-	-	ns	MUFA ↑ SFA ↓	-	Fiedorowicz-Szatkowska et al. [80]
High-tannin fava bean, rapeseed meal	100	-	-	-	-	ns	MUFA ↑ SFA — ns;	-	
Rapeseed meal, pea, yellow lupin	100	ns	ns	-	ns	ns	-	-	Zmudzińska et al. [18]
Rapeseed meal, pea	37	-	ns	-	ns	P ↑	SFA—ns PUFA ↑ MUFA—ns	-	Sońta et a. [79]
	52	-	ns	-	ns	ns	SFA— ns PUFA ↑ MUFA—ns	-	
	68	-	ns	-	ns	P ↑	SFA ↓ PUFA ↑ MUFA—ns	-	
	100	-	ns	-	ns	P ↑	SFA ↓ PUFA ↑ MUFA—ns	-	
Corn distiller’s dried grain with solubles, rapeseed oil	50	ns	ns	ns	↓	P↓ F ↑	SFA ↓ UFA ↑ MUFA—ns PUFA—ns	ns	Hanczakowska and Swiatkiewicz [82]
Corn distiller’s dried grain with solubles, beef tallow	50	ns	ns	ns	↓	P ↓ F ↑	SFA—ns UFA—ns MUFA—ns PUFA—ns	ns	
Corn distiller’s dried grain with solubles, coconut oil	50	ns	ns	ns	↓	P ns F ↑	SFA—ns UFA ↓ MUFA—ns PUFA ↓	Ns	
Sunflower meal, fava bean, potato protein	100	ns	ns	-	ns	ns	-	-	Mordenti et al. [99]
Spirulina	56	ns	ns	ns	ns	ns	ns	da-3 ↑	Martins et al. [91]
Chlorella vulgaris	20	ns	ns	ns	↓	ns	↑ PUFA	da- 8 ↑	Martins et al. [91]
Spirulina	100	ns	ns	ns	ns	ns	-	ns	Altmann et al. [7]
Hermetia illucens	100	ns	ns	ns	ns	ns	-	ns	
Hermetia illucens	18	ns	ns	ns	ns	F ↑	SFA—ns MUFA—ns PUFA—ns	-	Yu et al. [77]
	36	ns	ns	ns	ns	F ↑	SFA—ns MUFA—ns PUFA—ns	-	
Fish meal sileage	10	ns	ns	ns	ns	-	-	-	Cândido et al. [69]
	22	ns	ns	ns	ns	-	-	-	
	34	ns	ns	ns	ns	-	-	-	

^1^ “ns“ indicates that no statistical differences between control group (SB) and experimental diets (partial or total SB replacement) were observed; ^2^ “-” indicates that the parameter was not studied; ^3^ “↑” or “↓” indicate that SB replacement led to and increase or decrease in the parameter; ^4^ measured as Warner-Bratzler shear force except for Altmann et al. [7], who used MORS; ^5^ measured as drip and/or cooking loss.

## Data Availability

Data sharing not applicable.

## References

[1] Parisi G., Tulli F., Fortina R., Marino R., Bani P., Zotte A.D., De Angelis A., Piccolo G., Pinotti L., Schiavone A. (2020). Protein hunger of the feed sector: The alternatives offered by the plant world. Ital. J. Anim. Sci..

[2] Reckmann K., Blank R., Traulsen I., Krieter J. (2016). Comparative life cycle assessment (LCA) of pork using different protein sources in pig feed. Arch. Anim. Breed..

[3] Mungkung R., Aubin J., Prihadi T.H., Slembrouck J., van der Werf H.M., Legendre M. (2013). Life Cycle Assessment for environmentally sustainable aquaculture management: A case study of combined aquaculture systems for carp and tilapia. J. Clean. Prod..

[4] Nijdam D., Rood T., Westhoek H. (2012). The price of protein: Review of land use and carbon footprints from life cycle assessments of animal food products and their substitutes. Food Policy.

[5] Alexandratos N., Bruinsma J. (2012). World Agriculture towards 2030/2050: The 2012 Revision. ESA Working Papers.

[6] Lombardi G., Parrini S., Atzori R., Stefani G., Romano D., Gastaldi M., Liu G. (2021). Sustainable agriculture, food security and diet diversity. The case study of Tuscany, Italy. Ecol. Model..

[7] Altmann B.A., Neumann C., Rothstein S., Liebert F., Mörlein D. (2019). Do dietary soy alternatives lead to pork quality improvements or drawbacks? A look into micro-alga and insect protein in swine diets. Meat Sci..

[8] Van Zanten H.H.E., Bikker P., Meerburg B.G., de Boer I.J.M. (2017). Attributional versus consequential life cycle assessment and feed optimization: Alternative protein sources in pig diets. Int. J. Life Cycle Assess..

[9] Świątkiewicz M., Olszewska A., Grela E., Tyra M. (2021). The Effect of Replacement of Soybean Meal with Corn Dried Distillers Grains with Solubles (cDDGS) and Differentiation of Dietary Fat Sources on Pig Meat Quality and Fatty Acid Profile. Animals.

[10] Tallentire C.W., Mackenzie S.G., Kyriazakis I. (2018). Can novel ingredients replace soybeans and reduce the environmental burdens of European livestock systems in the future?. J. Clean. Prod..

[11] Wilkinson J.M., Lee M.R.F. (2018). Review: Use of human-edible animal feeds by ruminant livestock. Animal.

[12] Sońta M., Łukasiewicz-Mierzejewska M., Puppel K., Rekiel A., Więcek J., Batorska M. (2022). Influence of raw pea (*Pisum sativum*) or blue lupin seeds (*Lupinus angustifolius*) on the level of selected bioactive substances in pork meat. Ann. Anim. Sci..

[13] Mottet A., de Haan C., Falcucci A., Tempio G., Opio C., Gerber P. (2017). Livestock: On our plates or eating at our table? A new analysis of the feed/food debate. Glob. Food Secur..

[14] Schwediauer P., Hagmüller W., Zollitsch W. (2017). Germination of faba beans (*Vicia faba* L.) for organic weaning piglets. Org. Agric..

[15] Sardi L., Gastaldo A., Borciani M., Bertolini A., Musi V., Garavaldi A., Martelli G., Cavallini D., Nannoni E. (2020). Pre-Slaughter Sources of Fresh Meat Quality Variation: The Case of Heavy Pigs Intended for Protected Designation of Origin Products. Animals.

[16] Mazzocchi C., Orsi L., Zilia F., Costantini M., Bacenetti J. (2022). Consumer awareness of sustainable supply chains: A choice experiment on Parma ham PDO. Sci. Total Environ..

[17] Früh B., Schlatter B., Isensee A., Maurer V., Willer H. (2014). Report on Organic Protein Availability and Demand in Europe.

[18] Zmudzińska A., Bigorowski B., Banaszak M., Roślewska A., Adamski M., Hejdysz M. (2020). The Effect of Diet Based on Legume Seeds and Rapeseed Meal on Pig Performance and Meat Quality. Animals.

[19] Banaszak M., Kuźniacka J., Biesek J., Maiorano G., Adamski M. (2020). Meat quality traits and fatty acid composition of breast muscles from ducks fed with yellow lupin. Animal.

[20] Hejdysz M., Kaczmarek S., Rutkowski A. (2016). Extrusion cooking improves the metabolisable energy of faba beans and the amino acid digestibility in broilers. Anim. Feed Sci. Technol..

[21] Bocian M., Kapelański W., Adamowicz M., Jankowiak H., Cebulska A., Gimińska A., Mońko A. (2015). Influence of nutrition of pigs with legume-enriched mixtures on the quality of pork. Nauk. Przyr. Technol..

[22] Zaworska-Zakrzewska A., Kasprowicz-Potocka M., Wiśniewska Z., Rutkowski A., Hejdysz M., Kaczmarek S., Nowak P., Zmudzińska A., Banaszak M. (2020). The Chemical Composition of Domestic Soybean Seeds and the Effects of Partial Substitution of Soybean Meal by Raw Soybean Seeds in the Diet on Pigs’ Growth Performance and Pork Quality (M. Longissimus Lumborum). Ann. Anim. Sci..

[23] Atsbeha D.M., Flaten O., Olsen H.F., Kjos N.P., Kidane A., Skugor A., Prestløkken E., Øverland M. (2020). Technical and economic performance of alternative feeds in dairy and pig production. Livest. Sci..

[24] Kim S.W., Less J.F., Wang L., Yan T., Kiron V., Kaushik S.J., Lei X.G. (2019). Meeting Global Feed Protein Demand: Challenge, Opportunity, and Strategy. Annu. Rev. Anim. Biosci..

[25] Taliercio E., Kim S.W. (2013). Identification of a second major antigenic epitope in the α-subunit of soy β-conglycinin. Food Agric. Immunol..

[26] Kim S.W. (2010). Bio-fermentation Technology to Improve Efficiency of Swine Nutrition. Asian-Australas. J. Anim. Sci..

[27] Vohra K., Dureja H., Garg V. (2016). An Insight of Pulses: From Food to Cancer Treatment. J. Pharmacogn. Nat. Prod..

[28] Tuśnio A., Barszcz M., Taciak M., Święch E., Wójtowicz A., Skomiał J. (2021). The Effect of a Diet Containing Extruded Faba Bean Seeds on Growth Performance and Selected Microbial Activity Indices in the Large Intestine of Piglets. Animals.

[29] Degola L., Jonkus D. (2018). The Influence of Dietary Inclusion of Peas, Faba Bean and Lupin as a Replacement for Soybean Meal on Pig Performance and Carcass Traits. Agron. Res..

[30] Watson C.A., Reckling M., Preissel S., Bachinger J., Bergkvist G., Kuhlman T., Lindström K., Nemecek T., Topp C.F.E., Vanhatalo A., Sparks D.L. (2017). Chapter Four—Grain Legume Production and Use in European Agricultural Systems. Advances in Agronomy.

[31] Milczarek A., Osek M. (2016). 13. Partial Replacement of Soybean with Low-Tannin Faba Bean Varieties (Albus or Amulet): Effects on Growth Traits, Slaughtering Parameters and Meat Quality of Puławska Pigs. Ann. Anim. Sci..

[32] Sirtori F., Crovetti A., Acciaioli A., Pugliese C., Bozzi R., Campodoni G., Franci O. (2014). Effect of dietary protein level on carcass traits and meat properties of Cinta Senese pigs. Animal.

[33] Gatta D., Russo C., Giuliotti L., Mannari C., Picciarelli P., Lombardi L., Giovannini L., Ceccarelli N., Mariotti L. (2013). Influence of partial replacement of soya bean meal by faba beans or peas in heavy pigs diet on meat quality, residual anti-nutritional factors and phytoestrogen content. Arch. Anim. Nutr..

[34] Sobotka W., Fiedorowicz-Szatkowska E. (2021). The Effect of Replacing Genetically Modified Soybean Meal with 00-Rapeseed Meal, Faba Bean and Yellow Lupine in Grower-Finisher Diets on Nutrient Digestibility, Nitrogen Retention, Selected Blood Biochemical Parameters and Fattening Performance of Pigs. Animals.

[35] Hawthorne W. Pulses Nutritional Value and Their Role in The Feed Industry. https://www.semanticscholar.org/paper/pulses-nutritional-value-and-their-role-in-the-feed/06472aefdb19d49da89f7a15794096e757e5abbe.

[36] White G.A., Smith L.A., Haudijk J.G.M., Homer D., Kyriazakis I., Wiseman J. (2015). Replacement of soya bean meal with peas and faba beans in growing/finishing pig diets: Effect on performance, carcass composition and nutrient excretion. Anim. Feed Sci. Technol..

[37] Meng Z., Liu Q., Zhang Y., Chen J., Sun Z., Ren C., Zhang Z., Cheng X., Huang Y. (2021). Nutritive value of faba bean (*Vicia faba* L.) as a feedstuff resource in livestock nutrition: A review. Food Sci. Nutr..

[38] Angell A.R., Angell S.F., de Nys R., Paul N.A. (2016). Seaweed as a protein source for mono-gastric livestock. Trends Food Sci. Technol..

[39] Kumar N., Bansal A., Sarma G., Rawal R.K. (2014). Chemometrics tools used in analytical chemistry: An overview. Talanta.

[40] Skylas D.J., Paull J.G., Hughes D.G.D., Gogel B., Long H., Williams B., Mundree S., Blanchard C., Quail K.J. (2019). Nutritional and anti-nutritional seed-quality traits of faba bean (*Vicia faba*) grown in South Australia. Crop Pasture Sci..

[41] Smith L.A., Houdijk J.G.M., Homer D., Kyriazakis I. (2013). Effects of dietary inclusion of pea and faba bean as a replacement for soybean meal on grower and finisher pig performance and carcass quality1. J. Anim. Sci..

[42] Avilés-Gaxiola S., Chuck-Hernández C., Saldívar S.O.S. (2017). Inactivation Methods of Trypsin Inhibitor in Legumes: A Review. J. Food Sci..

[43] Multari S., Stewart D., Russell W.R. (2015). Potential of Fava Bean as Future Protein Supply to Partially Replace Meat Intake in the Human Diet. Compr. Rev. Food Sci. Food Saf..

[44] Pieper R., Taciak M., Pieper L., Święch E., Tuśnio A., Barszcz M., Vahjen W., Skomiał J., Zentek J. (2016). Comparison of the nutritional value of diets containing differentially processed blue sweet lupin seeds or soybean meal for growing pigs. Anim. Feed. Sci. Technol..

[45] Huang Y.F., Gao X.L., Nan Z.B., Zhang Z.X. (2017). Potential value of the common vetch (*Vicia sativa* L.) as an animal feedstuff: A review. J. Anim. Physiol. Anim. Nutr..

[46] Oghbaei M., Prakash J. (2016). Effect of primary processing of cereals and legumes on its nutritional quality: A comprehensive review. Cogent Food Agric..

[47] Kambashi B., Kalala G., Dochain D., Mafwila J., Rollin X., Boudry C., Picron P., Bindelle J. (2016). Nutritive value of three tropical forage legumes and their influence on growth performance, carcass traits and organ weights of pigs. Trop. Anim. Health Prod..

[48] Kaensombath L., Lindberg J.E. (2012). Effect of replacing soybean protein by taro leaf (*Colocasia esculenta* (L.) Schott) protein on growth performance of exotic (Landrace × Yorkshire) and native (Moo Lath) Lao pigs. Trop. Anim. Health Prod..

[49] Stødkilde L., Damborg V.K., Jørgensen H., Lærke H.N., Jensen S.K. (2019). Digestibility of fractionated green biomass as protein source for monogastric animals. Animal.

[50] Florou-Paneri P., Christaki E., Giannenas I., Bonos E., Skoufos I., Tsinas A., Tzora A., Peng J. (2014). Alternative Protein Sources to Soybean Meal in Pig Diets. J. Food Agric. Environ..

[51] Okrouhlá M., Stupka R., Čítek J., Šprysl M., Trnka M., Kluzáková E. (2008). Effect of lean meat proportion on the chemical composition of pork. Czech J. Food Sci..

[52] Sanjayan N., Heo J.M., Nyachoti C.M. (2014). Nutrient digestibility and growth performance of pigs fed diets with different levels of canola meal from Brassica napus black and *Brassica juncea* yellow. J. Anim. Sci..

[53] Yun H.M., Lei X.J., Lee S.I., Kim I.H. (2017). Rapeseed meal and canola meal can partially replace soybean meal as a protein source in finishing pigs. J. Appl. Anim. Res..

[54] Little K.L., Bohrer B.M., Maison T., Liu Y., Stein H.H., Boler D.D. (2015). Effects of feeding canola meal from high-protein or conventional varieties of canola seeds on growth performance, carcass characteristics, and cutability of pigs. J. Anim. Sci..

[55] Hasan I., Khan R.A., Alharbi W., Alharbi K.H., Abu Khanjer M., Alslame A. (2020). Synthesis, characterization and photo-catalytic activity of guar-gum-*g*-aliginate@silver bionanocomposite material. RSC Adv..

[56] Phengsavanh P., Lindberg J.E. (2013). Effect of replacing soybean protein with protein from porcupine joint vetch (*Aeschynomene histrix* BRA 9690) and stylo (*Stylosanthes guianensis* Composite) leaf meal on growth performance of native (Moo Lath) Lao pigs. Trop. Anim. Health Prod..

[57] Li Y., Chen L., Zhang Y., Wu J., Lin Y., Fang Z., Che L., Xu S., Wu D. (2018). Substitution of soybean meal with detoxified Jatropha curcas kernel meal: Effects on performance, nutrient utilization, and meat edibility of growing pigs. Asian-Australas. J. Anim. Sci..

[58] Yaakob Z., Ali E., Zainal A., Mohamad M., Takriff M.S. (2014). An overview: Biomolecules from microalgae for animal feed and aquaculture. J. Biol. Res..

[59] Makkar H.P.S., Tran G., Heuzé V., Giger-Reverdin S., Lessire M., Lebas F., Ankers P. (2016). Seaweeds for livestock diets: A review. Anim. Feed Sci. Technol..

[60] Corino C., Modina S.C., Di Giancamillo A., Chiapparini S., Rossi R. (2019). Seaweeds in Pig Nutrition. Animals.

[61] Øverland M., Mydland L.T., Skrede A. (2018). Marine macroalgae as sources of protein and bioactive compounds in feed for monogastric animals. J. Sci. Food Agric..

[62] Kibria S., Kim I.H. (2019). Impacts of dietary microalgae (*Schizochytrium* JB5) on growth performance, blood profiles, apparent total tract digestibility, and ileal nutrient digestibility in weaning pigs. J. Sci. Food Agric..

[63] García-Vaquero M., Brunton N., Lafarga T., Lafarga T., Acién G. (2021). Chapter 7—Microalgae as a Source of Pigments for Food Applications. Cultured Microalgae for the Food Industry.

[64] Yan L., Lim S.U., Kim I.H. (2012). Effect of Fermented Chlorella Supplementation on Growth Performance, Nutrient Digestibility, Blood Characteristics, Fecal Microbial and Fecal Noxious Gas Content in Growing Pigs. Asian-Australas. J. Anim. Sci..

[65] Colla E., Menegotto A.L.L., Kalschne D.L., da Silva-Buzanello R.A., Canan C., Drunkler D.A., Konur O. (2020). Chapter 32—Microalgae: A New and Promising Source of Food. Handbook of Algal Science, Technology and Medicine.

[66] Pecka-Kielb E., Zachwieja A., Mista D., Zawadzki W., Zielak-Steciwko A., Jacob-Lopes E., Zepka L.Q. (2017). Use of Corn Dried Distillers Grains (DDGS) in Feeding of Ruminants. Frontiers in Bioenergy and Biofuels.

[67] Wang H., Wang L.-S., Shi B.-M., Shan A.-S. (2012). Effects of dietary corn dried distillers grains with solubles and vitamin E on growth performance, meat quality, fatty acid profiles, and pork shelf life of finishing pigs. Livest. Sci..

[68] Caldas J.V., Hilton K., Mullenix G., Xuemei D., England J.A., Coon C.N. (2020). Corn distillers dried grains with solubles: Nutrient analysis, metabolizable energy, and amino acid digestibility in broilers. J. Appl. Poult. Res..

[69] Cândido R.S., Watanabe P.H., De Oliveira P.J.D., Angelim A.L., Siqueira A.D.F., Ximenes J.C.M., Normando L., Melo J.M., Freitas E.R. (2017). Meat quality and performance of pigs fed diets with fish silage meal. Pesqui. Agropecuária Bras..

[70] Veldkamp T., Vernooij A.G. (2021). Use of insect products in pig diets. J. Insects Food Feed.

[71] Chia S.Y., Tanga C.M., Osuga I.M., Alaru A.O., Mwangi D.M., Githinji M., Subramanian S., Fiaboe K.K.M., Ekesi S., van Loon J.J.A. (2019). Effect of Dietary Replacement of Fishmeal by Insect Meal on Growth Performance, Blood Profiles and Economics of Growing Pigs in Kenya. Animals.

[72] Gasco L., Finke M., Van Huis A. (2018). Can diets containing insects promote animal health?. J. Insects Food Feed.

[73] Barragan-Fonseca K.B., Dicke M., Van Loon J.J.A. (2017). Nutritional value of the black soldier fly (*Hermetia illucens* L.) and its suitability as animal feed—A review. J. Insects Food Feed.

[74] Veldkamp T., Bosch G. (2015). Insects: A Protein-Rich Feed Ingredient in Pig and Poultry Diets. Anim. Front..

[75] Spranghers T., Michiels J., Vrancx J., Ovyn A., Eeckhout M., De Clercq P., De Smet S. (2018). Gut antimicrobial effects and nutritional value of black soldier fly (*Hermetia illucens* L.) prepupae for weaned piglets. Anim. Feed Sci. Technol..

[76] Biasato I., Renna M., Gai F., Dabbou S., Meneguz M., Perona G., Martinez S., Lajusticia A.C.B., Bergagna S., Sardi L. (2019). Partially defatted black soldier fly larva meal inclusion in piglet diets: Effects on the growth performance, nutrient digestibility, blood profile, gut morphology and histological features. J. Anim. Sci. Biotechnol..

[77] Yu M., Li Z., Chen W., Rong T., Wang G., Li J., Ma X. (2019). Use of *Hermetia illucens* larvae as a dietary protein source: Effects on growth performance, carcass traits, and meat quality in finishing pigs. Meat Sci..

[78] Quander-Stoll N., Früh B., Bautze D., Zollitsch W., Leiber F., Scheeder M.R. (2021). Sire-feed interactions for fattening performance and meat quality traits in growing-finishing pigs under a conventional and an organic feeding regimen. Meat Sci..

[79] Sońta M., Rekiel A., Więcek J., Batorska M., Puppel K. (2021). Alternative Protein Sources vs. GM Soybean Meal as Feedstuff for Pigs—Meat Quality and Health-Promoting Indicators. Animals.

[80] Sobotka W., Stanek M., Fiedorowicz-Szatkowska E. (2012). Fattening performance and the nutritional value of meat from finishing pigs fed diets containing different sources of vegetable protein. J. Elem..

[81] Giuberti G., Gallo A., Masoero F. (2012). Plasma glucose response and glycemic indices in pigs fed diets differing in in vitro hydrolysis indices. Animal.

[82] Hanczakowska E., Świątkiewicz M. (2014). Legume Seeds and Rapeseed Press Cake as Replacers of Soybean Meal in Feed for Fattening Pigs. Ann. Anim. Sci..

[83] Izquierdo E.G., de Mercado de la Peña E., Fernández J.G., Almenar C.T., Fernández E.G., Sandín A.V., Elorrieta M.M., Pedrosa M.M., Nuez P.L., Górriz M.A.L. (2017). Sustitución de soja por guisante de invierno en dietas de cerdos pesados: Impacto productivo del nivel de inhibidores de proteasas. Inf. Tec. Econ. Agrar..

[84] De Quelen F., Brossard L., Wilfart A., Dourmad J.-Y., Garcia-Launay F. (2021). Eco-Friendly Feed Formulation and On-Farm Feed Production as Ways to Reduce the Environmental Impacts of Pig Production Without Consequences on Animal Performance. Front. Vet. Sci..

[85] Gómez Izquierdo E., Gomez-Raya L., de Mercado de la Peña E., Ciruelos J.J., Rauw W.M. (2020). Feed Efficiency Can Be Sustained in Pigs Fed with Locally Produced Narbon Vetch (*Vicia narbonensis* L.). Sustainability.

[86] Skoufos I., Tzora A., Giannenas I., Bonos E., Papagiannis N., Tsinas A., Christaki E., Florou-Paneri P. (2016). Dietary Inclusion of Rapeseed Meal as Soybean Meal Substitute on Growth Performance, Gut Microbiota, Oxidative Stability and Fatty Acid Profile in Growing-Fattening Pigs. Asian J. Anim. Vet. Adv..

[87] Torres-Pitarch A., Moset V., Ferrer P., Cambra-Lopez M., Hernández P., Coma J., Pascual M., Serrano P., Cerisuelo A. (2014). The inclusion of rapeseed meal in fattening pig diets, as a partial replacer of soybean meal, alters nutrient digestion, faecal composition and biochemical methane potential from faeces. Anim. Feed Sci. Technol..

[88] Seoni E., Battacone G., Kragten S.A., Dohme-Meier F., Bee G. (2020). Impact of increasing levels of condensed tannins from sainfoin in the grower–finisher diets of entire male pigs on growth performance, carcass characteristics, and meat quality. Animal.

[89] Rivero L.E., Caro Y., Fernández L.A., Ayala L., Rivero A., Tamayo Y. (2020). Comportamiento Productivo de Cerdos En Ceba Alimentados Con Follaje Fresco de *Morus Alba* Como Sustituto Parcial Del Concentrado. REDVET.

[90] Šimkus A., Šimkiene A., Černauskienė J., Kvietkutė N., Černauskas A., Paleckaitis M., Kerzienė S. (2013). The Effect of Blue Algae Spirulina Platensis on Pig Growth Performance and Carcass and Meat Quality. Vet. Zootech..

[91] Martins C.F., Pestana J.M., Alfaia C.M., Costa M., Ribeiro D.M., Coelho D., Lopes P.A., Almeida A.M., Freire J.P.B., Prates J.A.M. (2021). Effects of *Chlorella vulgaris* as a Feed Ingredient on the Quality and Nutritional Value of Weaned Piglets’ Meat. Foods.

[92] Martins C.F., Pestana Assunção J., Ribeiro Santos D.M., Madeira M.S.M.D.S., Alfaia C.M.R.P.M., Lopes P.A.A.B., Coelho D.F.M., Cardoso Lemos J.P., de Almeida A.M., Mestre Prates J.A. (2020). Effect of dietary inclusion of Spirulina on production performance, nutrient digestibility and meat quality traits in post-weaning piglets. J. Anim. Physiol. Anim. Nutr..

[93] Huang B., Shi H., Wang L., Wang L., Lyu Z., Hu Q., Zang J., Li D., Lai C. (2021). Effects of Defatted Rice Bran Inclusion Level on Nutrient Digestibility and Growth Performance of Different Body Weight Pigs. Animals.

[94] Casas G.A., Overholt M.F., Dilger A.C., Boler D.D., Stein H.H. (2018). Effects of full fat rice bran and defatted rice bran on growth performance and carcass characteristics of growing-finishing pigs. J. Anim. Sci..

[95] DiGiacomo K., Leury B.J. (2019). Review: Insect meal: A future source of protein feed for pigs?. Animal.

[96] Meyer S., Gessner D.K., Braune M.S., Friedhoff T., Most E., Höring M., Liebisch G., Zorn H., Eder K., Ringseis R. (2020). Comprehensive evaluation of the metabolic effects of insect meal from Tenebrio molitor L. in growing pigs by transcriptomics, metabolomics and lipidomics. J. Anim. Sci. Biotechnol..

[97] Trabue S.L., Kerr B.J., Scoggin K.D., Andersen D., van Weelden M. (2020). Swine diets impact manure characteristics and gas emissions: Part II protein source. Sci. Total Environ..

[98] Rauw W.M., Gomez-Raya L., Martín-Pedrosa M., Sanz-Calvo M.A., de Mercado de la Peña E., Ciruelos J.J., Gómez-Izquierdo E. (2021). Replacing soybean meal with Narbon vetch (*Vicia narbonensis* L.) in pig diets: Composition of subcutaneous fat and fresh loin and sensory attributes of dry-cured product. Span. J. Agric. Res..

[99] Mordenti A.L., Martelli G., Brogna N., Nannoni E., Vignola G., Zaghini G., Sardi L. (2012). Effects of a soybean-free diet supplied to Italian heavy pigs on fattening performance, and meat and dry-cured ham quality. Ital. J. Anim. Sci..

